# Supplementary material for: Synergetic electrode architecture for efficient graphene-based flexible organic light-emitting diodes
Source: Nat Commun. 2016 Jun 2;7:11791. doi: 10.1038/ncomms11791 (PMC4895731; doi:10.1038/ncomms11791)
Supplement: Supplementary Information — Supplementary Figures 1-11, Supplementary Tables 1-6, Supplementary Notes 1-3 and Supplementary References. [file ncomms11791-s1.pdf]

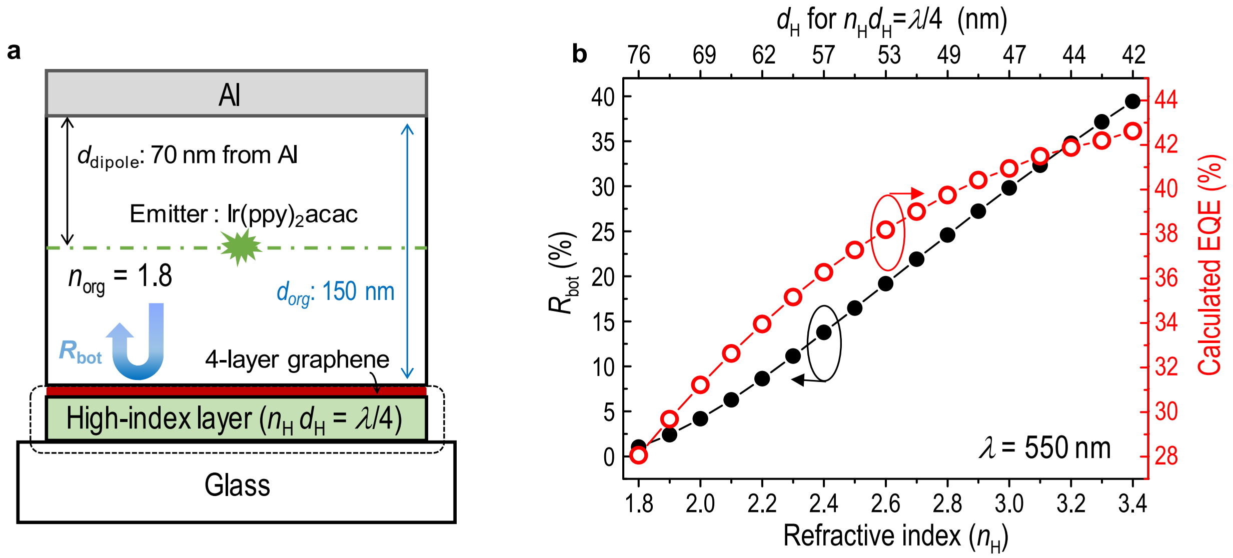

Supplementary Figure 1. The effect of the refractive index (n_H) of the high-index under-layer on the reflectance from the bottom anode (R_{bot}) and the EQE of an OLED. (a) A schematic diagram of a device structure for optical simulation. The optical thickness of high-index layer is set as $\lambda/4$. **(b)** The calculated R_{bot} (black filled symbols; left-axis) and EQE (red open circles; right-axis) of graphene based OLEDs in a configuration of glass/ high-index layer ($d_H= \lambda/4n_H$)/organic layer (150 nm)/ Al as a function of n_H. Dipole emitter of Ir(ppy)$_2$acac is assumed to be located 70 nm away from the organic/ Al interface. λ is set at 550 nm.

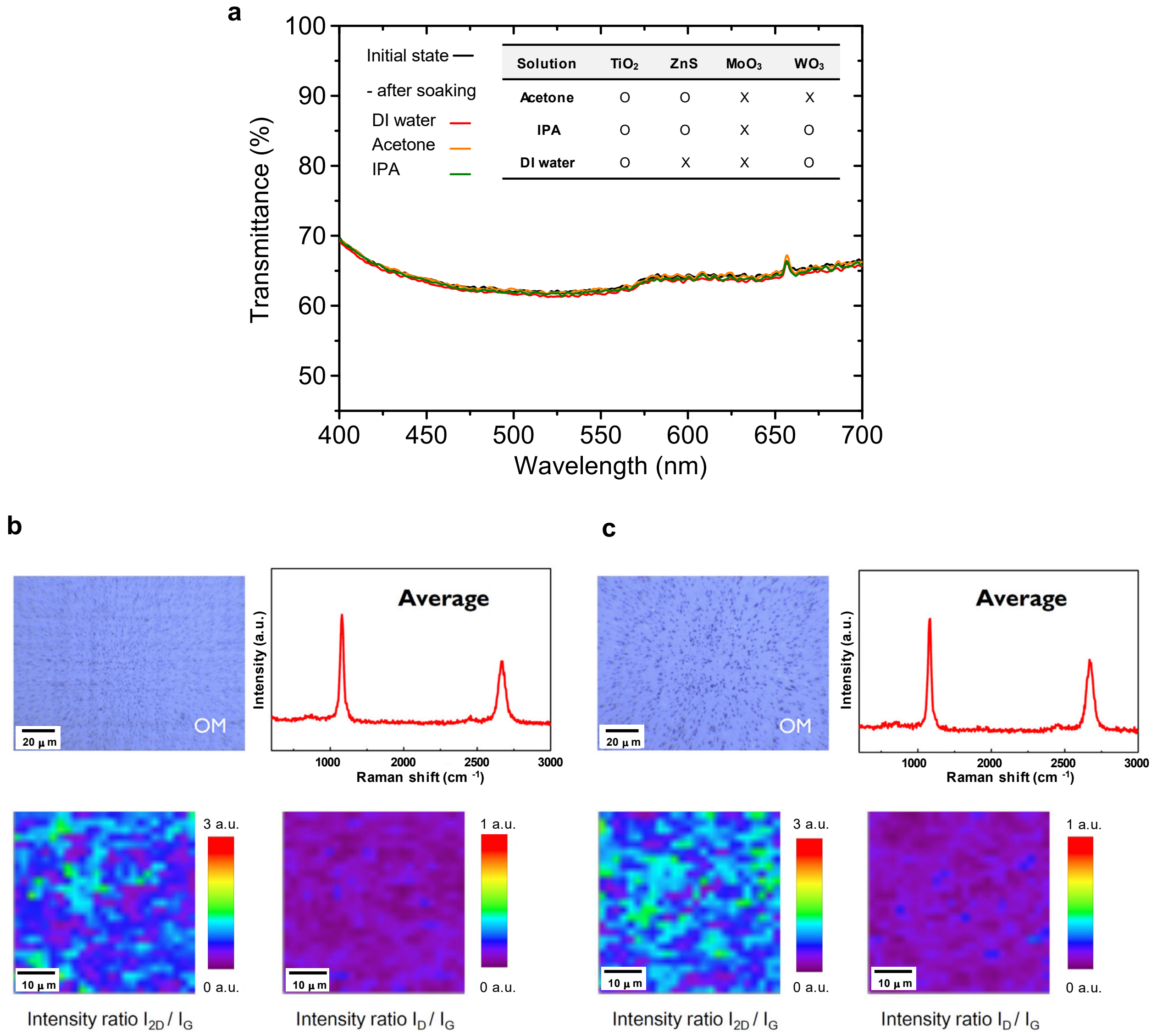

Supplementary Figure 2. Compatibility of TiO_2 with graphene-transfer process and the quality of graphene electrodes transferred to TiO_2 layers. (a) Transmittance of TiO_2 on glass substrates before and after being soaked in DI water, acetone, and isopropyl alcohol (IPA). Inset: table showing the test results for other high-index materials tried. **(b)** Optical microscopy images and Raman data for multilayer graphene (MLG) on a glass substrate. **(c)** Optical microscopy images and Raman data for multilayer graphene (MLG) on a TiO_2-coated glass substrate.

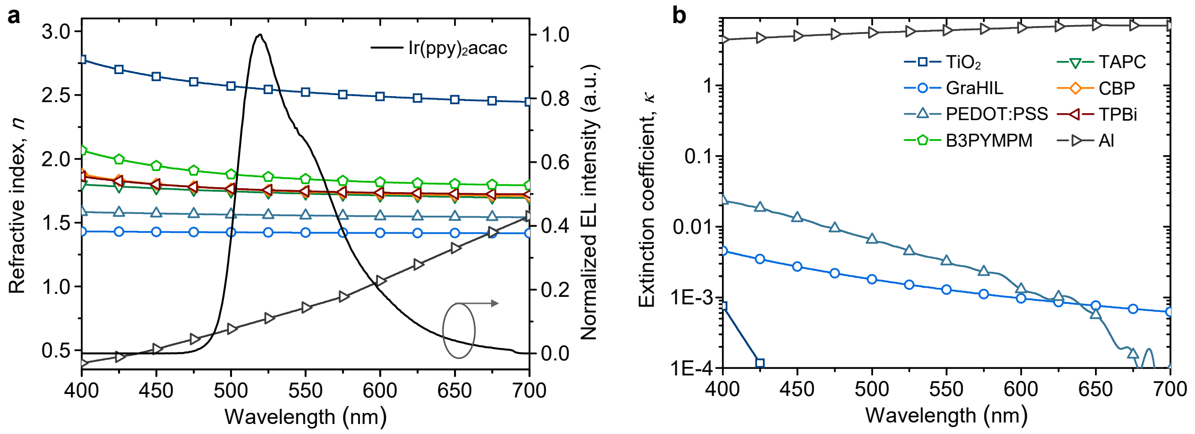

Supplementary Figure 3. Optical properties of materials used in this work. (a) The refractive indices (*n*) and **(b)** extinction coefficients (*κ*) of materials used. In (a) the normalized emission spectrum of Ir(ppy)$_2$acac emitters is also shown.

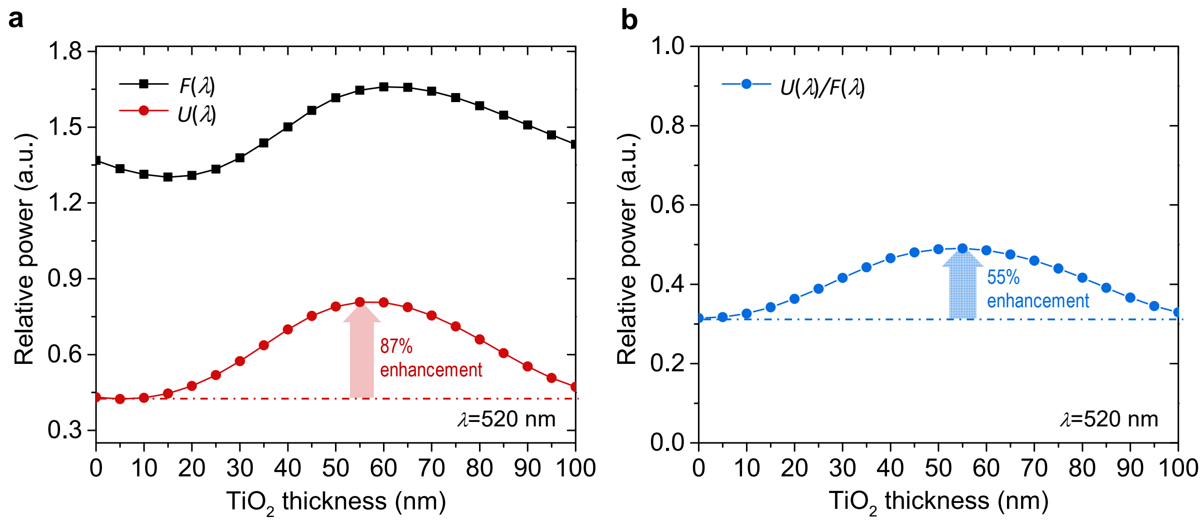

Supplementary Figure 4. Importance of including Purcell effect in optical simulation. (a) *F*(λ), *U*(λ), and **(b)** *U*(λ)/*F*(λ) vs TiO_2 thickness in OLED devices with the proposed TiO_2/Graphene (4-layer)/GraHIL (70 nm) electrodes, where *F*(*λ*) and *U*(*λ*) are Purcell factor and the normalized power radiated to the outside of a given cavity, respectively, at a given *λ*. Outcoupling ratio is given by *U*(*λ*)/ *F*(*λ*). Monochromatic emitter (*λ* = 520 nm) was assumed in this calculation for simplicity. Summary of the calculation results and detailed discussion are provided in Supplementary Table 1 and Supplementary Note 1.

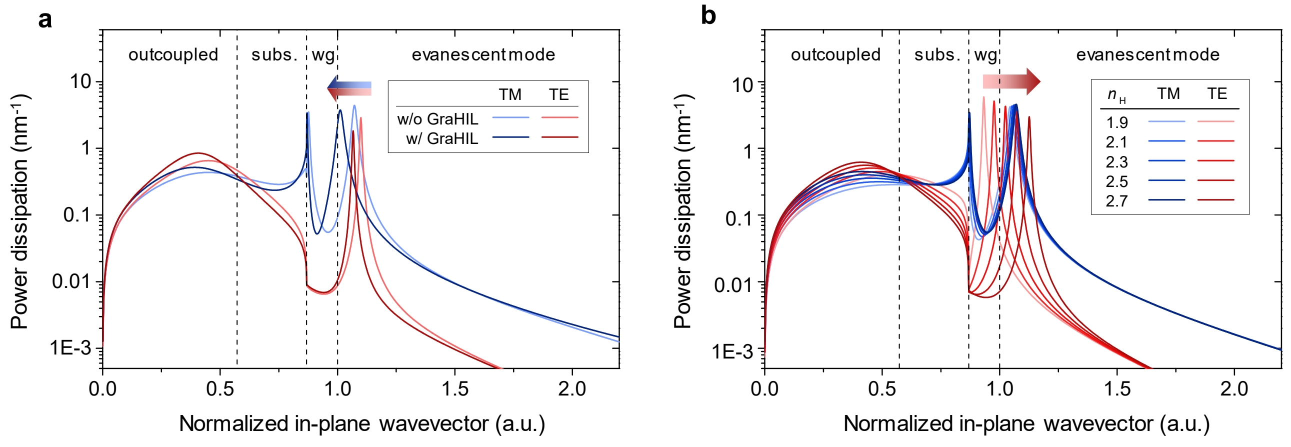

Supplementary Figure 5. Power distribution of graphene-based OLEDs under study vs. normalized in-plane wave vector – comparison between transverse-electric (TE) and transverse-magnetic (TM) waves. (a) Effect of presence of a low-index hole-injection layer in OLEDs with TiO_2 (55 nm)/graphene electrodes. **(b)** Effect of the refractive index (n_H) of a high-index under-layer (H) in OLEDs with H /graphene electrodes in which the thickness of H (= d_H) is set at $d_H = \lambda/(4n_H)$. λ is set at 550 nm. The relative power fractions coupled to each mode in graphene-based OLEDs are shown in Supplementary Table 2 for the anode structures under study. In (a) and (b), it can be noted that (i) the low-index HIL shifts the evanescent modes of TM waves toward lower normalized in-plane wave vector; and that (ii) the high-index under-layer enhances outcoupled modes by resonant enhancement but tends to push back the waveguided modes of TE waves toward evanescent modes. The latter can be reclaimed to some degree upon combinatory use of low-index HILs, emphasizing the synergetic role of both high-index under-layers and low-index HILs.

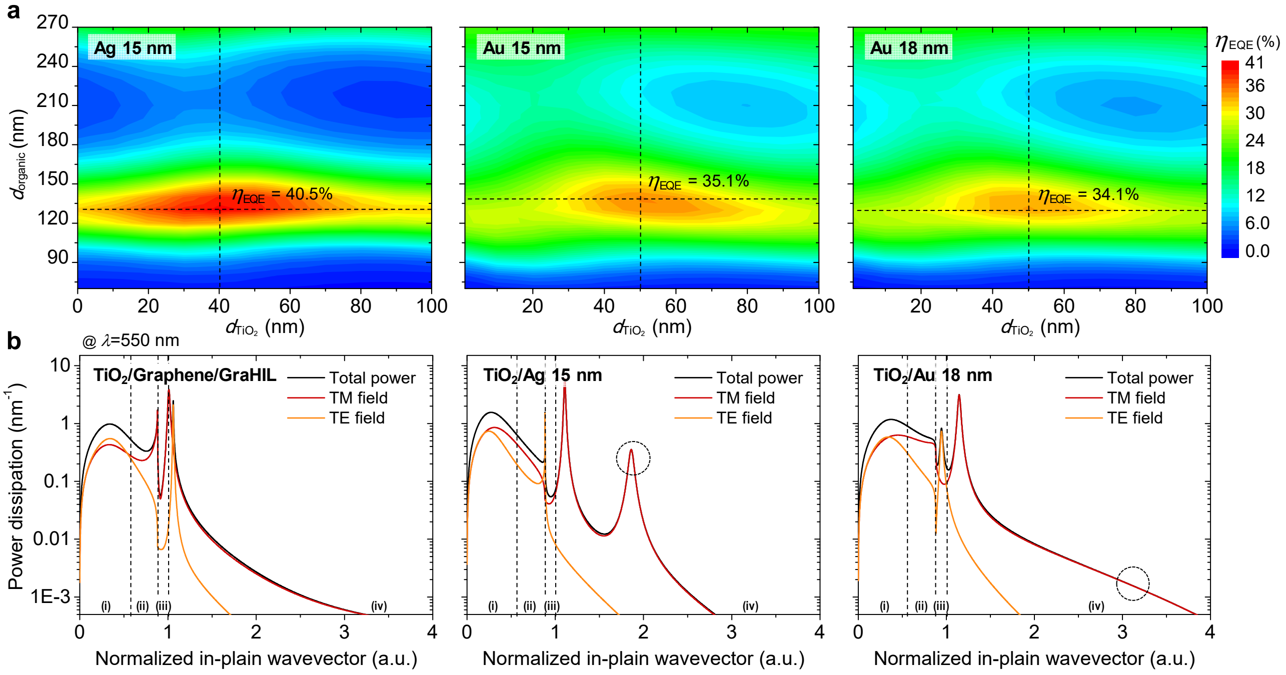

Supplementary Figure 6. The comparison of TiO_2/Graphene/GraHIL based OLEDs (metal-free cavity) with TiO_2/M-based OLEDs (metal-based cavity) where M = Ag (15 nm) or Au (15 or 18 nm). (a) and **(b)** The calculated EQE and relative power distribution vs. normalized in-plane wave vector of OLED devices under study. (see Supplementary Table 3 for device structure). In (b), (i)-(iv) correspond to air-, substrate-, waveguided-, and evanescent modes, respectively, and the thicknesses of organic layers and TiO_2 layers were set at the optimal values obtained in (a). In case of TiO_2/Graphene/GraHIL based devices, the related calculation results are shown in Fig. 1d and Fig. 2b in the main manuscript. The optical constants of thin gold and silver layers were borrowed from the literature[1,2]. The presence of surface plasmon polariton (SPP) modes due to a thin metal film is known to be found in large normalized in-plane wave vectors (= u) (typically larger than 1) for transverse magnetic (TM) fields. Such a mode is clearly observed in devices with Ag; in case of devices with Au, it is not as apparent, but one can still see the long tail that extends to u of ca. 4. Considering the absorption within the transparent electrodes, the distribution of power corresponding to each mode is summarized in Supplementary Table 3. One can clearly note the superiority of the OLEDs with the proposed electrode to those based on conventional, dielectric-capped thin-metal electrodes. That is, air modes as well as summation of air and substrate modes are highest for the OLED devices with TiO_2/Graphene/GraHIL electrodes. Significance of loss to SPP/evanescent modes and absorption can also be noted for metal-based cavity.

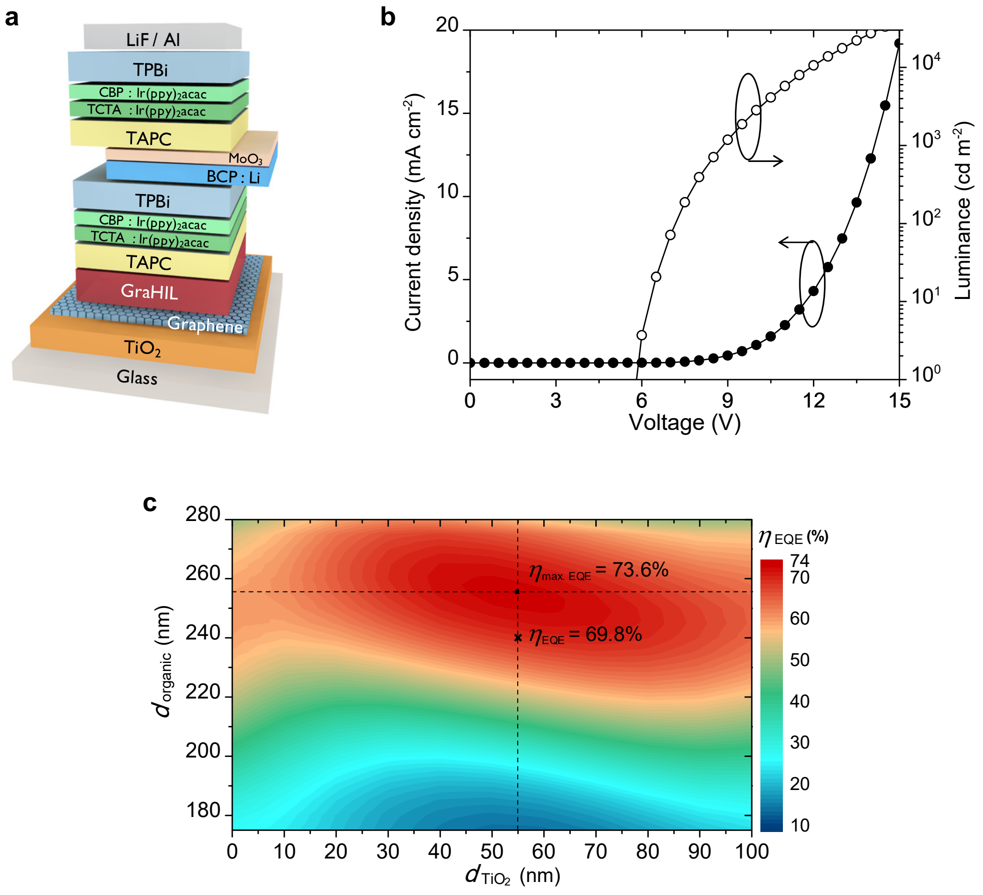

Supplementary Figure 7. Application of the proposed electrodes to multi-junction OLEDs. (a) The schematic structure of the multi-junction OLEDs employing the proposed TiO_2/ Graphene/ GraHIL electrodes. **(b)** Current density-voltage-luminance characteristics of the proposed multi-junction OLED. **(c)** Contour plot of the calculated EQE vs. total organic layer thickness ($d_{organic}$) and TiO_2 thickness (d_{TiO2}). The results of optical simulation are presented in comparison with experimental data in Supplementary Table 4. Detailed description of multi-junction devices is provided in Supplementary Note 2.

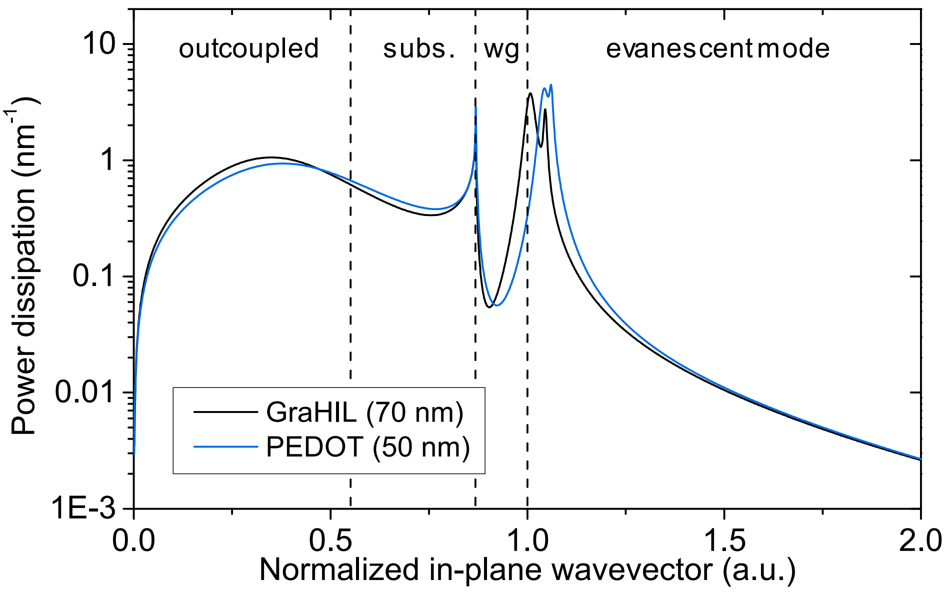

Supplementary Figure 8. The effectiveness of reducing SPP modes in OLEDs with the low index hole-injection layer. Power distribution for each mode in TiO_2 / graphene-based OLEDs having low-index HILs of GraHIL (n_L=1.42) or PEDOT:PSS (n_L=1.56). (λ =550 nm). The calculated power fractions of each mode with GraHIL or PEDOT:PSS as a low index hole-injection layer are represented in Supplementary Table 5.

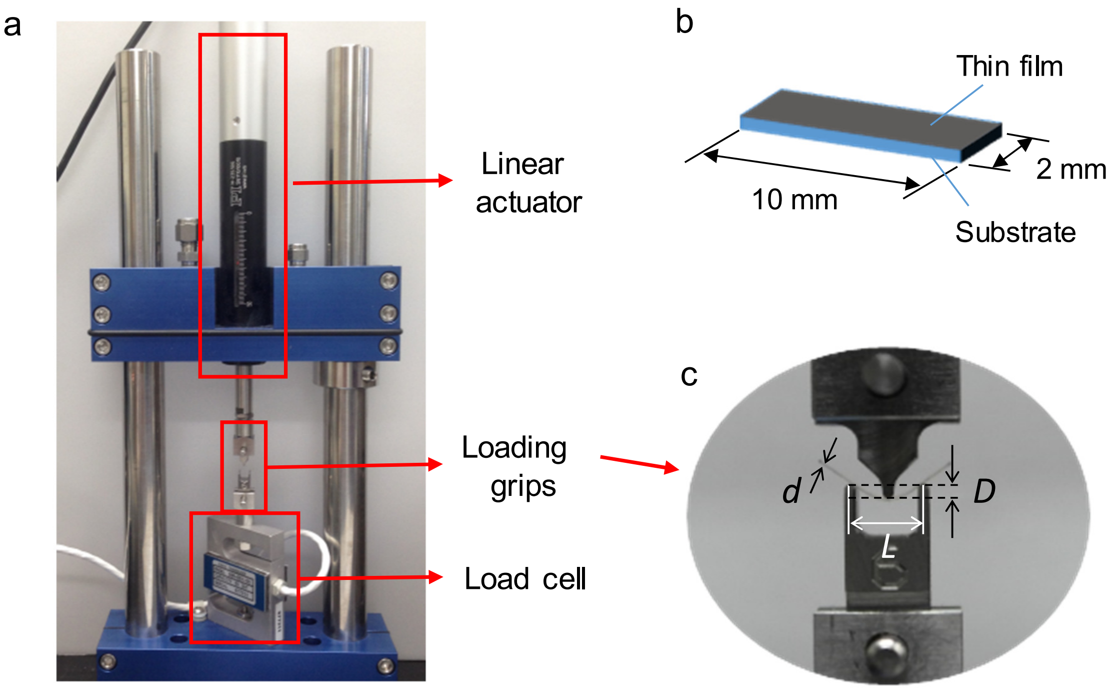

Supplementary Figure 9. The 3-point loading test system and specimens for flexural strain. (a) A photograph of the 3-point loading test system. The system consists of a linear actuator, loading grips, and a load cell. **(b)** Dimensions of each specimen used in this work. **(c)** Photographs of 200 μm-thick PET specimens with 5.0% flexural strain and definition of geometrical parameters important for control of flexural strain (ε_f). D is maximum deflection of the center of the specimen, d is the thickness of a specimen tested, and L is support span. The relation of ε_f to D, d, and L is provided in Figure 5 in the main text. In this test, each specimen was inserted in the loading grips and verified as contacted with no strain by 0.01 N of load from load cell. All tests were conducted with a constant displacement rate of 5 μm s^{-1}.

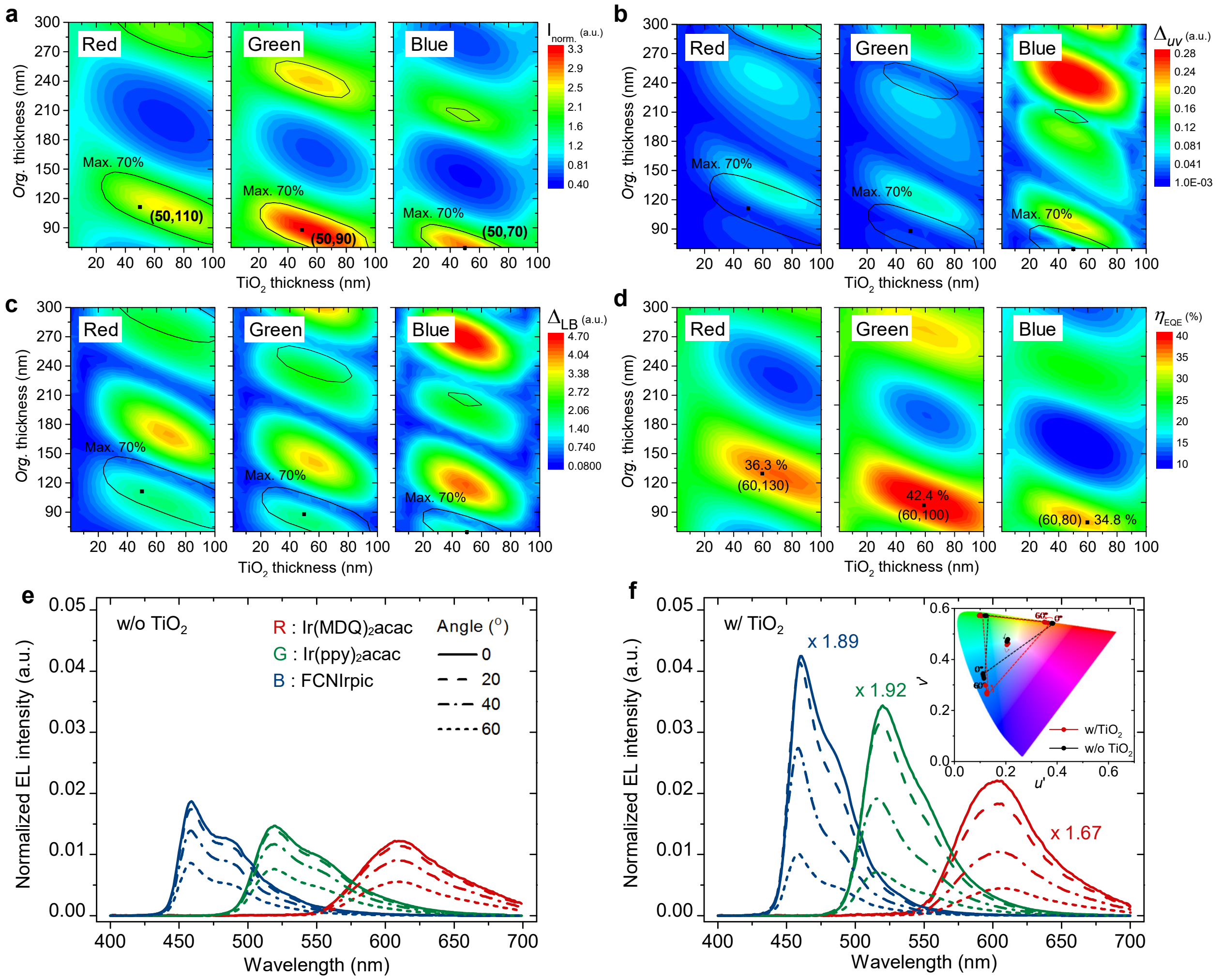

Supplementary Figure 10. Optical design of the proposed R,G, and B OLEDs based on the proposed electrodes with a common TiO_2 layer for both angular color stability and high efficiency. (a)-(d) The calculated normal intensity ($I_{norm.}$), angular spectral shift (Δ_{uv}), angular intensity deviation (Δ_{LB}) and external quantum efficiency (η_{EQE}) as a function of stacked organic and TiO_2 thickness. The black solid lines represent the range of 70% intensity from maximum value. (Red emitter: $Ir(MDQ)_2acac$, green emitter: $Ir(ppy)_2acac$ and blue emitter: FCNIrpic[3,4]). **(e)-(f)** The calculated normalized electro-luminescence intensity vs. wavelength for several viewing angles: (e) without a TiO_2 layer; (f) with a 50-nm-thick common TiO_2 (inset: The angular color shift of each sub-pixel color and synthesized white color on CIE 1976 *u*'*v*' chromaticity diagram.). Definition of the terms used here and summary of calculation results are provided in Supplementary Note 3 and Supplementary Table 6.

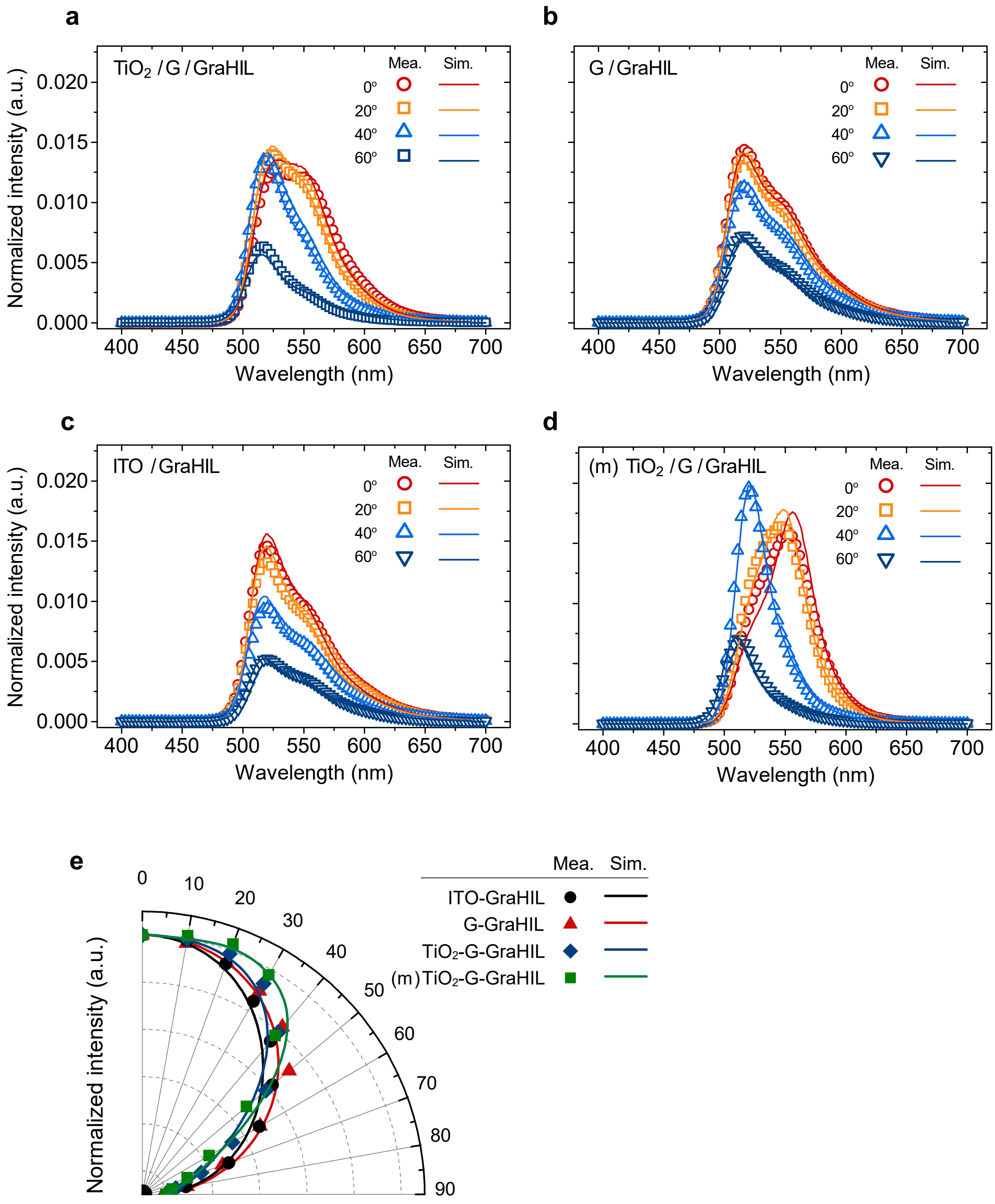

Supplementary Figure 11. Quantitative agreement between experimental and optical simulation results. (a) to **(d)** Normalized electro-luminescence spectrum of devices with electrodes of (a) TiO_2/Graphene(G)/GraHIL (b) Graphene(G)/GraHIL (c) ITO/GraHIL. That of a multi-junction device with TiO_2/Graphene(G)/GraHIL is also shown in (d). **(e)** Angular luminous intensity characteristics of the OLEDs under study. '(m)' indicates the data obtained from the multi-junction device.

Supplementary Table 1. The comparison of simulation models on the enhancement ratio of the efficiency of a cavity-based OLED device [=Device 2] to that of a non-cavity control OLED device [=Device 1*].

	**"Simplified" model** $U^{(2)}(\lambda_0)/U^{(1)}(\lambda_0)$	**"Full" model** $[U^{(2)}(\lambda_0)/F^{(2)}(\lambda_0)]/ [U^{(1)}(\lambda_0)/F^{(1)}(\lambda_0)]$
Efficiency Enhancement Ratio (λ_0=520 nm)**	1.87	1.55

* Device structure under comparison:

Device 1 (control dev.): Glass/Graphene (4-layer)/GraHIL (70 nm)/OS1/Al
Device 2: Glass/TiO_2 (55 nm) /Graphene (4-layer)/GraHIL (70 nm)/OS1/Al

** Both devices are assumed to have a monochromatic light emitter at λ_0 of 520 nm.

Supplementary Table 2. Relative power fractions coupled to each mode in graphene-based OLEDs with different anode structures.

Unit : %

Anode types	HIL types	Outcoupled mode	Substrate mode	W/G mode	Evanescent mode	Absorption	Other losses
TiO_2/Graphene		43.4	16.5	6.7	22.4	8.9	2.1
Graphene	w/ GraHIL	31.5	29.3	21.1	9.1	6.7	2.3
ITO		28.9	22.6	16.0	16.4	13.8	2.3
TiO_2/Graphene		37.7	18.7	3.9	30.9	6.6	2.2
Graphene	w/o GraHIL [a)]	24.2	34.9	6.8	25.2	6.6	2.3
ITO		22.9	25.8	11.9	24.3	12.8	2.3

[a)] The optical thickness of organic layers was adjusted for the same total optical thickness to meet Fabry-Perot cavity resonance condition.

Supplementary Table 3. The calculated power fraction of each mode in OLEDs under study.

Unit : %

Device structure* (*with optimized TiO_2 and organic thickness)	Air mode	Substrate mode	Absorption	W/G mode	Evanescent mode	Other losses
Glass/ TiO_2 (60 nm)/ Graphene GraHIL (70 nm)/ Organic layer (90 nm) / Al	43.9	17.1	9.4	6.1	21.5	2.0
Glass/ TiO_2 (40 nm)/ Ag (15 nm)/ Organic layer (130 nm)/ Al	40.5	13.0	16.3	1.0	27.4	1.8
Glass/ TiO_2 (50 nm)/ Au (15 nm)/ Organic layer (140 nm)/ Al	35.1	20.1	16.1	5.4	21.6	1.7
Glass/ TiO_2 (50 nm)/ Au (18 nm)/ Organic layer (130 nm)/ Al	34.1	16.5	18.1	5.5	24.1	1.7

Supplementary Table 4. The summary of measured efficiency and optical simulation data of TiO_2/Graphene/GraHIL based multi-junction OLEDs under study.

Unit: %

Measured (Max. value)		Calculation					
		EQE (this work)			EQE (optically optimal)		
**Device**	EQE	total	bottom	top	total	bottom	top
**Tandem**	62.1	69.8	37.8	32.0	73.6	39.9	33.7
**Tandem** w/ a lens	103.3	109.8*	57.6	52.2	110.5	58.4	52.1

* Theoretical EQE values for devices with a half-ball lens are obtained by adding air modes and substrate modes assuming the lens can extract all the substrate modes.

Supplementary Table 5. Relative power fraction coupled to each mode in TiO_2/graphene-based OLEDs according to low-index HILs.

Unit: %

HIL types	Out-coupled mode	Substrate mode	W/G mode	Evanescent mode	Absorption	Other losses
GraHIL (70 nm)	43.4	16.5	6.7	22.4	8.9	2.1
PEDOT:PSS (50 nm)	40.2	18.0	3.4	28.6	7.7	2.1

Supplementary Table 6. The summary of angular emission characteristics (Δ_{uv}, Δ_{LB}, and $\Delta_{uv}^{(white)}$) of the R,G,B OLEDs with the proposed electrodes in which a common TiO_2 layer is used for all R,G,B subpixels.

TiO_2 (nm)	Sub-pixel color	d_{org} (nm)	$\Delta_{uv}^{30°}$	$\Delta_{uv}^{60°}$	Δ_{LB}	$I_{norm.}$ (a.u.)	f_{white}* (a.u.)	$\Delta_{uv}^{30°(white)}$	$\Delta_{uv}^{60°(white)}$
0	Red	110	0.0017	0.0039	0.011	1.38	2.09	0.0073 (1.83 JNCD)	0.0085 (2.13 JNCD)
	Green	90	0.0034	0.0062	0.013	1.77	1.00		
	Blue	70	0.0112	0.0197	0.026	1.58	2.32		
50**	Red	110	0.0013	0.0056	0.186	2.31	1.98	0.0127 (3.17 JNCD)	0.0176 (4.40 JNCD)
	Green	90	0.0066	0.0067	0.172	3.40	1.00		
	Blue	70	0.0293	0.0332	0.163	3.00	2.08		

* f_{white} : the intensity mixing ratio of each color to get white color having (0.33, 0.33) of CIE 1931 coordinates.

** The thickness of a common TiO_2 layer that can achieve the best angular color stability while the forward intensity (I_{norm}) is maintained at least within 70% of the maximum achievable value.

Supplementary Note 1. The importance of including Purcell effect in optical simulation of OLEDs

Importance of including Purcell effect in optical simulation of OLEDs has been emphasized by several authors[5-8]. This is because a dipole emitter in an OLED is not in free space but in stratified multilayer media including a metal layer and often forms a microcavity structure, influencing its radiative decay process. In spite of its importance, however, simplified models that do not account for Purcell effect (hereafter 'Simplified Models') have often been used, and the full optical model correctly accounting for Purcell effect (hereafter 'Full Model') has been used only by a few groups partly because of the complexity in its formulations. In addition to its correct account of changed radiative decay rate, incorporation of Purcell effect in Full Model inherently takes account of dissipation/ coupling of the power generated by an emitting dipole into various loss channels present in OLEDs (waveguide mode, surface plasmon polartion (SPP) mode).

In this section, let us illustrate the importance of using full model in quantitatively assessing the change in efficiency (as well as other emission characteristics) when a device structure is varied. Within Full Model, the key parameter at a given wavelength of λ is Purcell factor $F(\lambda)$. It is the ratio of radiative decay rate of a dipole emitter within a cavity to that of a dipole emitter in free space. Note that $F(\lambda)$ is also the total radiated power of a dipole emitter within the cavity normalized to that of a dipole emitter in free space. $F(\lambda)$ then equals to the summation of (normalized) power coupled or dissipated to air mode, substrate-confined mode, waveguide mode, and SPP/evanescent mode in an OLED. The air mode is given as $U(\lambda)$, which is the normalized power radiated to outside of the given cavity. It is noteworthy that the outcoupling ratio at λ is given by $U(\lambda)/F(\lambda)$ instead of $U(\lambda)$ itself. External quantum efficiency (EQE) and power efficiency (PE) are thus given by the following equations[7].

$$\begin{aligned}\eta_{\mathrm{EQE}} &= \gamma_{\mathrm{elec}}\int s(\lambda)\eta^{*}_{\mathrm{rad}}(\lambda)\eta_{\mathrm{out}}(\lambda)d\lambda \\ &= \gamma_{\mathrm{elec}}\int s(\lambda)\eta^{*}_{\mathrm{rad}}(\lambda)\frac{U(\lambda)}{F(\lambda)}d\lambda \ \rightarrow \gamma_{\mathrm{elec}}\int s(\lambda)\frac{U(\lambda)}{F(\lambda)}d\lambda \quad \text{if } \eta_{\mathrm{rad}}\approx 1\end{aligned}$$

where $\eta^{*}_{\mathrm{rad}}(\lambda)=\dfrac{\eta_{\mathrm{rad}}F(\lambda)}{(1-\eta_{\mathrm{rad}})+\eta_{\mathrm{rad}}F(\lambda)}$, Supplementary Equation 1

γ_{elec}, $s(\lambda)$, and η^{*}_{rad} being normalized electrical efficiency, normalized source spectrum, and radiative quantum efficiency within the cavity structure, respectively.

$$\begin{aligned}\eta_{\mathrm{PE}} &= \frac{\gamma_{\mathrm{elec}}}{eV_{\mathrm{bias}}}K_{\mathrm{m}}\int f_{\mathrm{L}}(\lambda)\frac{hc}{\lambda}s(\lambda)\eta^{*}_{\mathrm{rad}}(\lambda)\eta_{\mathrm{out}}(\lambda)d\lambda \\ &= \frac{\gamma_{\mathrm{elec}}}{eV_{\mathrm{bias}}}K_{\mathrm{m}}\int f_{\mathrm{L}}(\lambda)\frac{hc}{\lambda}s(\lambda)\eta^{*}_{\mathrm{rad}}(\lambda)\frac{U(\lambda)}{F(\lambda)}d\lambda \\ &\rightarrow \frac{\gamma_{elec}}{eV_{\mathrm{bias}}}K_{\mathrm{m}}\int f_{\mathrm{L}}(\lambda)\frac{hc}{\lambda}s(\lambda)\frac{U(\lambda)}{F(\lambda)}d\lambda \quad \text{if } \eta_{\mathrm{rad}}\approx 1\end{aligned}$$

Supplementary Equation 2

where $K_m = 683\,\mathrm{lm\,W^{-1}}$, V_{bias}, e, and $f_{\mathrm{L}}(\lambda)$ are applied voltage, electronic charge, and photopic response curve, respectively.

For simplicity, let's consider a monochromatic case ($\lambda=\lambda_0$) with η_{rad} close to unity. In this case,

$$s(\lambda)=\delta(\lambda-\lambda_0)$$

$$\eta_{\mathrm{EQE}}\approx\gamma_{\mathrm{elec}}\frac{U(\lambda_0)}{F(\lambda_0)},\quad \eta_{\mathrm{PE}}\approx\frac{\gamma_{\mathrm{elec}}}{eV_{\mathrm{bias}}}\left(\frac{hc}{\lambda_0}\right)K_{\mathrm{m}}f_{\mathrm{L}}(\lambda_0)\frac{U(\lambda_0)}{F(\lambda_0)}$$

Supplementary Equation 3

That is, both EQE and PE scale with $U(\lambda_0)/F(\lambda_0)$. As can be seen in Supplementary Fig. 4. shown below, both $U(\lambda_0)$ and $F(\lambda_0)$ vary depending on a device structure particularly when the degree of cavity resonance changes. Therefore, it is critical to monitor $U(\lambda_0)$ and $F(\lambda_0)$ (and of course $U(\lambda_0)/F(\lambda_0)$) to quantitatively account for enhancement ratio between the efficiencies of two devices under comparison, especially if the two devices have a different degree of cavity resonance. In Simplified Model, however, the effect of Purcell factor is not included, and thus looks at only the change in $U(\lambda)$ when comparing two

devices. As can be seen in Supplementary Fig. 4 and Supplementary Table 1, Simplified Model tends to predict an efficiency enhancement ratio different from (often larger than) what Full Model predicts. Simplified Models may serve as a quick guide to predict efficiency enhancement, spectral change, etc., but a correct quantitative assessment should be done with Full Model.

Supplementary Note 2. Optical analysis of multi-junction OLEDs based on the proposed transparent electrode structure.

Multi-junction OLED technology adopts a tandem structure stacking individual unit devices with charge-generation layers (CGLs) connecting those unit devices[9]. It aims at boosting EQE by effectively doubling the number of generated photons per carrier injected. The improved EQE in this way is particularly important for long device lifetime because it allows for low-current-operation for a given brightness. The detailed layer composition of the device used in this work (Supplementary Fig 7a.) is as follows:

TiO_2 (55 nm)/ 4-layer-graphene/ GraHIL (70 nm)/ TAPC (15 nm)/ TCTA:$Ir(ppy)_2acac$ (5 nm)/ CBP:$Ir(ppy)_2acac$ (5 nm)/ TPBi (60 nm)/ [BCP:Li (20 nm, 10% doping)/ MoO_3 (5 nm)]/ TAPC (65 nm)/ TCTA:$Ir(ppy)_2acac$ (5 nm)/ CBP:$Ir(ppy)_2acac$ (5 nm)/ TPBi (60 nm)/ LiF (1 nm)/ Al (150 nm) where BCP:Li / MoO_3 serves as a CGL.

One may recall that EQE in multi-junction OLEDs can ideally be twice as large as that of a single-junction OLED; such results have been observed in ITO-based OLEDs with little cavity resonance effect. The following question naturally arises: is it possible to double the EQE of OLEDs the structure of which is already optimized for maximal efficiency by exploiting the cavity resonance phenomena?

The calculation results summarized shown below indicates that it is also possible but with the EQE values expected for 2nd-order cavity structures, instead of the EQE value expected for 1st-order cavity structure. This is why the measured value is not as high as 2×40%, in which 40% EQE is from the 1st-order cavity structure.

From the optical perspectives, the bottom unit in the multi-junction cell is equivalent to a 2nd-order cavity structure where its emission zone is located at 2nd antinode from the cathode, and the top unit is equivalent to that where its emission zone is located at 1st antinode from the cathode. As shown in Supplementary Table 4, the EQE values in the optimized case for those 2nd-order cavity OLEDs are 39.9 % and 33.7%, respectively, summation of which will lead to the total EQE of 73.6%. Because the best optical structure requires a relatively thick organic layer, however, it is likely to induce resistive power loss. (see Supplementary Fig. 7c) The present multi-junction device is thus slightly off from the best optical structure, and thus the EQE values for the aforementioned 2nd-order cavity OLEDs are 37.8 % and 32.0%, leading to the total EQE of 69.8%. The experimentally observed EQE value of 62.1% is consistent with the calculation results with the electrical balance factor set at 0.89.

Supplementary Note 3. Design of graphene-based RGB OLED subpixels: common TiO_2-layer-based approach.

Let us design R,G,B subpixel OLED stacks based on TiO_2/Graphene/GraHIL electrodes. We will try to find a design that exhibits both high efficiency and minimal angular color shift with a common TiO_2 layer for all pixels. For this calculation, TiO_2/Graphene (4 layers) /GraHIL (50 nm) based devices with phosphorescent emitters (R : $Ir(MDQ)_2acac$, G : $Ir(ppy)_2acac$, and B : FCNIrpic[3,4]) were used. The normal intensity (I_{norm}) , angular color shift (Δ_{uv}) in CIE 1976 (u',v') color coordinate, and standard deviation of angular intensity pattern from that of Lambertian (Δ_{LB}) were calculated using the same advanced classical electromagnetic theory employed in the manuscript. Definition of these terms are as follows:

$$\Delta_{uv}(\theta) = \sqrt{(u'(\theta) - (u'(0^\circ))^2 + (v'(\theta) - (v'(0^\circ))^2}, \quad 0^\circ < \theta \leq 60^\circ$$
$$\Delta_{uv} = max(\Delta_{uv}(\theta)), \quad \Delta_{LB} = \sqrt{(\sum_{i=1}^{n}[I(\theta_i)/I(0^\circ) - \cos\theta_i]^2)/n}$$

Supplementary Equation 4

For the angular color shift, values within 60 degrees (with respect to a direction normal) were monitored; industrial convention used for the personal portable displays typically takes care of the values within 30 degrees, and so the calculation in this work looks over a wider range of observation angle. To make the RGB synthesized white stable over observation angle, it is critical to (i) minimize Δ_{uv} for each R, G, B, respectively; and to (ii) make Δ_{LB} values similar for R,G,B so that $I(\theta)$'s of R,G,B drop at the same rate over θ to maintain the white balance when the viewing angle is changed.

From the contour plots shown in Supplementary Figure 10, one can easily tell that the design based on a 50 nm-thick common TiO_2 layer can lead to a still significant enhancement in forward intensity and EQE vs. non-cavity counterparts and at the same time, Δ_{uv} on the order of a few JNCD, where JNCD refers to 'just noticeable color difference' and corresponds to Δ_{uv} of 0.004.[10] (See Supplementary Table 6 for summary of the results.) Color shift of a few JNCD is hardly recognized by average human eyes, and thus displays with angular color shift of a few JNCD over 30 degrees are generally regarded as 'excellent' in display industry. Of course, a design aiming at maximal efficiency can also be made with a common TiO_2 layer at the expense of color stability. The design based on a 60 nm-thick common TiO_2 layer is such an example. (Supplementary Fig. 10d.)

Supplementary References

1. Liu, X. *et al.* The design of ZnS/Ag/ZnS transparent conductive multilayer films. *Thin Solid Films* **441**, 200-206 (2003).

2. Wang, Z. *et al.* Unlocking the full potential of organic light-emitting diodes on flexible plastic. *Nature. Photon.* **5**, 753-757 (2011).

3. Shin, H. *et al.* Blue phosphorescent organic light-emitting diodes using an exciplex forming co-host with the external quantum efficiency of theoretical limit. *Adv. Mater*. **26**, 4730-4734 (2014).

4. Jeon, S. *et al.* External quantum efficiency above 20% in deep blue phosphorescent organic light-emitting diodes. *Adv. Mater*. **23**, 1436-1441 (2011).

5. Wasey, J. A. E. *et al.* Efficiency of spontaneous emission from planar microcavities. *J. Mod. Opt*. **47**, 725-741 (2000).

6. Brütting, W. *et al*. Device efficiency of organic light-emitting diodes: progress by improved light outcoupling. *Phys. Status Solidi A* **210**, 44-65 (2013).

7. Furno, M., Meerheim, R., Hofmann, S., Lüssem, B., Leo, K. Efficiency and rate of spontaneous emission in organic electroluminescent devices. *Phys. Rev. B*. **85**, 115205 (2012).

8. Kim, S-Y. *et al.* Organic light-emitting diodes with 30% external quantum efficiency based on a horizontally oriented emitter. *Adv. Funct. Mater.* **23,** 3896-3900 (2013).

9. Chiba, T. *et al.* Ultra-high efficiency by multiple emission from stacked organic light-emitting devices. *Org. Electron*. **12**, 710-715 (2011).

10. Steckel, J. S. *et al.* Quantum dots: The ultimate down-conversion material for LCD displays. *J. Soc. Inf. Disp.* **23**, 294-305 (2015).
